# Biosynthesis of ZnONP Using *Chamaecostus cuspidatus* and Their Evolution of Anticancer Property in MCF-7 and A549 Cell Lines

**DOI:** 10.3390/nano12193384

**Published:** 2022-09-27

**Authors:** Menaka Priya Balaji, Rajakumar Govindasamy, Naiyf S. Alharbi, Shine Kadaikunnan, Muthu Thiruvengadam, Venkidasamy Baskar, Vijayarangan Devi Rajeswari

**Affiliations:** 1 Department of Biomedical Sciences, School of Bioscience and Technology, VIT, Vellore 632114, Tamil Nadu, India; 2 Department of Orthodontics, Saveetha Dental College and Hospitals, Saveetha Institute of Medical and Technical Sciences (SIMATS), Saveetha University, Chennai 600077, Tamil Nadu, India; 3 Department of Botany and Microbiology, College of Science, King Saud University, Riyadh 11451, Saudi Arabia; 4 Department of Crop Science, College of Sanghuh Life Sciences, Konkuk University, Seoul 05029, Korea; 5 Department of Oral and Maxillofacial Surgery, Saveetha Dental College and Hospitals, Saveetha Institute of Medical and Technical Sciences (SIMATS), Chennai 600077, Tamil Nadu, India

**Keywords:** zinc oxide, *Chamaecostus cuspidatus*, antioxidant, anticancer

## Abstract

The ZnO nanoparticle synthesis using the leaf part of *Chamaecostus cuspidatus* was characterized using UV–Vis spectrophotometry, IR, XRD, DLS, FESEM, EDX, TEM, AFM and XPS. The MTT assay was used to examine the cytotoxicity activity against lung epithelial and breast cell lines, and the IC_50_ value was determined. The presence of ZnO nanoparticles, which range in size from 200 to 800 nm, was confirmed by the absorption peak at 350 nm. The median particle size was 145.1 nm, and the ζ -the potential was −19.45 mV, showing that ZnONP is stable. Zinc, carbon, and oxygen contribute to the elemental composition of ZnONP, as determined by EDX analysis. MTT assay was used to investigate in vitro cytotoxicity in MCF-7 and A549 cell lines. The cytotoxicity activity IC_50_ value was determined to be 30 μg/mL for the A549 cell line and 37 μg/mL for the MCF-7 cell line.

## 1. Introduction

Nanotechnology has gained wide acceptance due to its superior properties and vast surface area. Molecular biology, inorganic and organic chemistry, material science, and physics are several diverse fields covered by nanotech [1]. Because of its nano-size (10^−9^) [2,3], shape (rod, cylinder, hexagonal, spherical, etc.), and high surface-to-volume ratio (40 times > particle), nanotechnology is now ubiquitously used in sensors, medical devices, catalysis, optical devices, DNA labelling, and drug delivery [4,5]. A “green combination” uses natural resources to synthesis nanocrystals [6]. Natural biological systems are employed to produce nanoscale particles such as zerovalent metallic nanoparticles by utilising a redox mechanism and secondary metabolites with functional groups that enhance the reduction and stability capabilities. These metabolites can bind to metal ions and form them into nanoparticles [7]. It is a more environmentally acceptable and less harmful way than any other. Sustainable construction, resource efficiency, biocompatible chemicals, durability, and safety are all advantages of integrating green chemistry and nanotechnology [8]. Therefore, it is essential for biosynthesis that offers compatibility for pharmacological and biomedical applications [9].

Furthermore, toxic organic substances are more frequently adsorbed on the surface of nanoparticles during chemical synthesis, resulting in adverse consequences for people when used in medical applications. The physical method necessitates the use of sophisticated instruments and is exorbitant. Although specific physical and chemical approaches for nanomaterial synthesis exist [10,11], this method is preferred since it produces fewer pollutants and is comparatively affordable [2]. More narrowly, herbs are the most biological and physiological substrates used for the environmentally friendly development of nanomaterials containing metabolic ions [12,13]. It could be because the botanical substrate is perceived to be more economically sound, highly treatable, and less unsafe than microorganisms [14,15]. The biomedical synthesis of the biomimetic method shows catalytic activity and functions as a stabilizer, reductant, plus capping agent in the crystallization process to hinder nanoparticle accumulation [6]. Some approaches and attributes that can affect a nanocrystal’s composition and purity include plant classification, parts of the plants, plant constituents, cellular enzymes, reaction duration, pH, heating rate, sample preparation, precursor concentration, centrifugation, and separation [16,17]. As a result, suitable parameters provide control and the exact synthesis of nanoparticles of the precise size and shape [18].

*Chamaecostus cuspidatus*, often known as the insulin plant, is an ethnopharmacological plant that grows primarily in tropical areas such as Indonesia and India. *C. cuspidatus* is indeed antidiabetic and antilipidemic [19]. It has concentrations of active ingredients such as flavonoids and phenolic acids, which aid in the stabilization and reduction of nanoparticles [20,21]. Antioxidants are plant chemicals that neutralize ROS and free radicals, which are also chelating metals. Metals are designed and constructed to progressively distribute metal ions in the cell [22]. Among all metals [7], zinc is the second most biological trace element [23,24], which is found in significant amounts in the brain, muscle, and bone [25]. It performs as a cofactor for several enzymes that contribute to intracellularly synthesizing biological macromolecules [23]. It is amphoteric, which implies special features such as UV filtering in cosmetics and skin care products, semiconducting, antimicrobial, and photocatalytic activity [26]. Zinc oxide nanoparticles (ZnONP) have been used for tissue growth, implantation, bioimaging, wound repair, and the creation of oncology medicines, among other applications in biomedical engineering [27]. Biosynthesized ZnO nanoparticles were found to have a high level of action against malignant cells and pathogenic microorganisms [28]. Additionally, zinc oxide nanoparticles exhibit cytotoxicity against cancerous cells without harming normal cells by inducing death in malignant cells by increasing ROS production. The release of Zn^2+^ ions from ZNONP is rapid in cancerous cells due to its slightly acidic pH. The results show that ZnONP displays pH-responsive cytotoxicity. Furthermore, it increased cellular internalization, raised oxidative stress, increased p53 activation, decreased caspase 3 and apoptosis, and lowered migrating rate, mitochondrial membrane potential, and decreased cell viability. Administration of ZnONP finally results in mitotic death, total cell death, necrosis, and extrinsic and intrinsic apoptosis in cancer cells [29]. With the aim to assess its contribution, the plant extract of *C. cuspidatus* was used as a reducing agent for the preparation of ZnONP. The standard characterization technique, as well as antioxidant, and cytotoxicity analysis, were used to investigate its properties.

## 2. Materials and Methods

The medicinal plant *C. cuspidatus* was collected in November 2021 from Alagiri in Vellore district, Tamil Nādu, India. The herbal sample was taxonomically identified (Authorization number: PARC/2022/4736) by Prof. P. Jayaraman, Director of Plant Anatomy Research Centre (PARC), West Tambaran, Chennai.

### 2.1. Collection of Plant Material and Preparation of Leaf Extracts

*C. cuspidatus* leaf material was separated, cleaned with deionized water, and dried in the dark for about 15 days. The leaves were smashed into a coarse powder using a blender. The leaf powder was stored in a dry, airtight container away from direct sunlight [3]. About 5 g of dry powder was mixed with 100 mL of milli-Q water and kept in a magnetic stirrer for 45 min. The herbal extraction was cooled to 37 °C before being filtered multiple times to remove any remaining waste or debris. Furthermore, all unconsolidated components were removed from the plant extract by centrifugation at 5000 rpm for 10 min (2×), and the resultant extract was kept at 4 °C for future use.

### 2.2. Green Synthesis of ZnONP Using C. cuspidatus

The biosynthesis of ZnONP followed a previously described procedure with minimal modifications. In order to prepare ZnONP, 0.9468 g of (0.1 M) zinc nitrate (Molecular weight: 189.36) in 50 mL of solution was mixed with 50 mL of herbal extract, and then it was homogeneously mixed [8]. The mixture pH was maintained by 1 mL sodium hydroxide, and the mixture was concentrated by keeping it at 60 °C [26]. The reaction color changed to a white-yellow precipitate, confirming ZnONP production [30]. The solution was cooled to ambient temperature before being centrifuged for 10 min at 5000 rpm (2×). The resulting supernatant residue was washed twice with milli-Q water. The probable ZnONP was washed in ethanol and dried using the hot plate technique. To remove moisture, pale yellow ZnONP was calcined at 300 °C in a muffle furnace [31]. Furthermore, ZnONP was characterized by microscopic and spectroscopic techniques.

### 2.3. Characterization of ZnONP

UV–Vis, IR, XRD, EDX, SEM, DLS, TEM, AFM and XPS were all used to characterise the ZnONP. 0.1 g ZnONP was dissolved in 10 mL distilled water and sonicated for 5 min to analyze. In overview, UV spectrophotometry (200–800 nm) JASCO (V-670 PC) was used to investigate zinc ions’ optical properties and bio-reduction to ZnONP. FTIR (IR-affinity-1, SHIMAZDU, Japan 2011) was used to identify the FTIR spectra of the powdered sample of the synthesis metal oxide nanoparticle and their active functional groups and stretching and bending vibrating in the ZnONP at the region of 450–4500 cm^−1^. Furthermore, DLS was used to determine the particle size of produced ZnONP, and ζ-potential measurements were used to examine the reliability of ZnONP. The X-ray diffractometer (Bruker, D8 Advanced) of angle 2ϴ in the range 20–80° with CuKα radiation (λ = 1.5406Ả). The morphology and crystalline nature of the ZnONP were confirmed using X-ray diffraction analysis [3]. XRD measures the symmetry, size, and shape of ZnONP. The morphology, microstructure, and elemental configuration of the synthesized ZnONP were carried out using scanning electron microscope (SEM) analysis instrument. The elemental composition of the ZnONP was investigated using energy dispersive X-ray analysis (EDX). TEM (transmission electron microscopy) analysis (FEI TECHNAI T20) was carried out to provide information on the shape and size of the distribution of synthesized ZnONP [31]. The analysis of atomic force microscopy (AFM) was used to detect nanoparticle size and roughness. From tip-correlated AFM, the size of the metal nanoparticles was seen. Following a 3 × 3 μm^2^ scan, the AFM result demonstrated a 2D and 3D view of the sample surface [32]. Using Nanosurf Easyscan 2, the surface topography of the thin films was examined (Model no. 23-06-154; manufactured by Nanosuf AGG Switzerland). At room temperature, images were captured. For ZnONP, the surface roughness root mean square (RMS) values were calculated over a 3 × 3 μm^2^ area. The chemical composition was evaluated by X-ray photoelectron spectroscopy (XPS) analysis, ESCALAB 250XI (Thermo Fischer Scientific Inc., Waltham, MA, USA) equipped with a monochromatic X-ray Al Kα radiation (1486.6 eV). The XPS spectra were collected at a take-off angle of 45° for the electron energy analyzer and the spot size was 200 μm. Pass energy (PE) of 20 eV was used for the high-energy resolution spectra (Zn 2p, O 1s, and C 1s). The spectra were analyzed using the CASA-XPS software [33].

### 2.4. Antioxidant Activity of ZnONP

The DPPH free radical scavenging experiment was used to determine ZnONP antioxidant properties. The antioxidant properties of DPPH are assumed to be due to their ability to donate hydrogen and become a stable diamagnetic molecule [34]. In short, 500 μL of 1M DPPH (2,2′-diphenyl-1-picrylhydrayl) solution with 100% methanol was added to various concentrations of ZnONP (5–50 μg/mL). The combination was thoroughly mixed before incubating in the dark for 30 min. The positive control used was ascorbic acid. The absorbance of the reactant was measured at OD_517 nm_ after incubation. The free radical scavenging potential was calculated using the formula.

Scavenging activity (%) = [(A_control_ − A_sample_)/A_control_] × 100

### 2.5. Cell Culture

Breast and lung cancer cells (MCF-7 and A549) were used to evaluate the cytotoxicity and MCF-10A as a control [35] against ZnONP. MCF-10A (non-tumorigenic human breast epithelial cell line), MCF-7 (Michigan Cancer Foundation-7), and A549 (Hypotriploid alveolar basal epithelial cell) were received from NCSS (National Centre for Cell Science, Pune, India). In a humidified environment, the cells were kept in minimal essential media (MEM) supplemented with 10% foetal bovine serum (FBS), penicillin (100 U/mL), and streptomycin (100 μg/mL). At 37 °C, the cells were seeded in a temperature-controlled environment containing 5% CO_2_ and 95% atmosphere [36]. The cells were examined for viability using the Trypan blue exclusion assay according to the instructions before being used in the experiments. The study included cells with a viability of over 95%.

### 2.6. MTT Assay Determination

The microculture tetrazolium test was used to determine the % cell viability using the following methodology [27,37,38]. In brief, the cells (1 × 10^4^) were cultured in 96-well culture plates and permitted to adhere for 24 h at 37 °C in high-humidity surroundings with 5% CO_2_ and 95% O_2_. The cells were subsequently treated to various concentrations of ZnONP (5–50 μg/mL) for 24 and 48 h (cisplatin was used as standard). MTT (5 mg/mL stock in PBS) was mixed (10 μL cell suspension) after exposure, and the plate was incubated for 4 h. The solution mixture was removed at the end of the incubation period, and 200 μL of DMSO was added to each well and gently mixed [39]. The plates were shaken for 10 min at 37 °C, and the absorbance was measured with a multiwell microplate scanner at 550 nm. Untreated sets were also prepared under identical conditions and served as the controls. The calculation was carried out using the IC_50_ value:

Cell Viability = [Absorbance of sample/Absorbance of control] × 100

### 2.7. Analytical Statistics

ANOVA was used to analyze the data, followed by Tukey’s HSD assessment. A *p*-value < 0.5 was considered statistically significant.

## 3. Results

The ZnONP synthesized using *C. cuspidatus* was characterized, and the results are presented below.

### 3.1. Visual Observation of ZnONP Synthesized by C. cuspidatus

**Figure 1:**
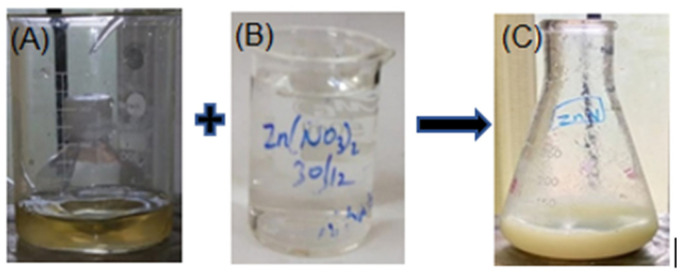
Synthesis of ZnONP. (**A**) *C. cuspidatus* leaf extract, (**B**) zinc nitrate, (**C**) ZnONP.

The extract filtrate color turned from dark green to pale yellow to a colorless liquid by stirring the *C. cuspidatus* leaf extract with an aqueous Zinc nitrate solution. Zinc oxide particles were formed when the green extract turned pale yellow (pH 10) at 60 °C. As illustrated in Figure 1, zinc nitrate ions were reduced by the bioactive compounds, resulting in white ZnONP precipitate [40]. The color shift recognized the ZnONP.

### 3.2. Characterization of ZnONP

#### 3.2.1. UV Spectrophotometry

**Figure 2:**
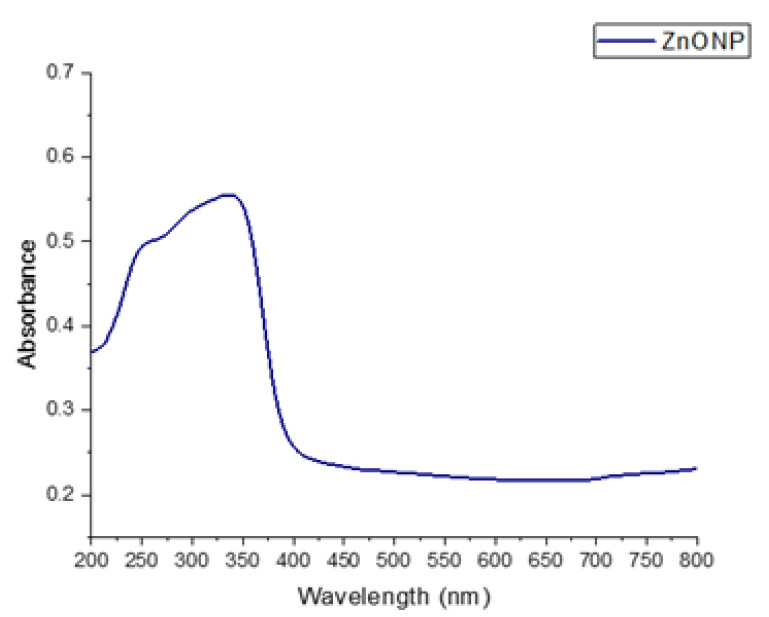
UV–Visible spectroscopy of synthesized ZnONP.

A UV–Visible spectrophotometer was used to examine the optical characteristics of ZnO. The presence of ZnONP nanocrystals was confirmed by absorption bands at 350 nm, as illustrated in Figure 2. Consequently, these were ZnONP. The UV–Vis absorption curve of ZnO nanoparticles is presented. Zinc oxide production was validated by finding the peak max near 280–380 nm [34]. As a result, the absorption spectra of ZnONP exhibited a pronounced blue shift, indicating that these metal nanoparticles should not be larger than the exciton Bohr radius.

#### 3.2.2. FT-IR Spectrum

**Figure 3:**
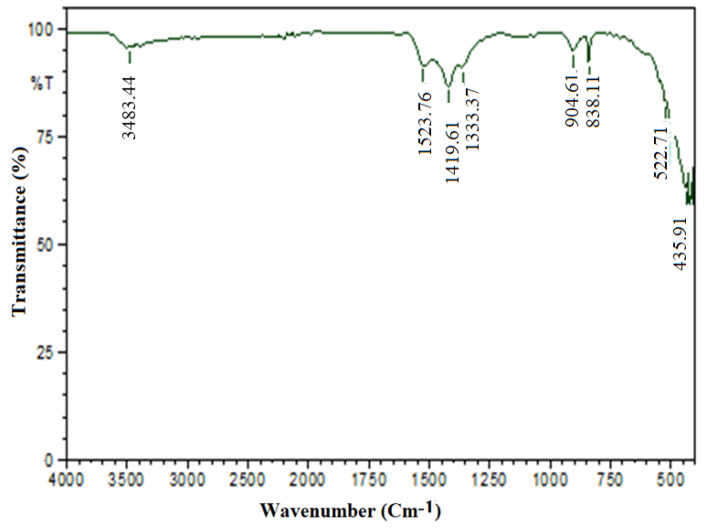
FTIR spectrum of synthesized ZnONP.

These nanoparticles were analyzed using FT-IR to identify the various active functional groups in the produced ZnONP nanocrystals. It helps to discover the functional and potential phytochemical compounds involved in the reduction and stabilization of ZnONP by giving an impression of the vibrational and rotational modes of existent molecules. The stretching vibration of the Zn–O peak appeared in the range of 400–800 cm^−1^ [41]. Other peak bonds confirmed the functional groups adsorbed on the surface of ZnONP. The functional group of ZnONP was denoted using the peaks [3]. According to Figure 3, the samples had absorption peaks of 3483.44, 1523.76, 1419.61, 1363.67, 904.61, 839.03, 522.71, and 435.91. The absorption peak at 3483.44 corresponded to dimeric O–H stretch, and 1523 corresponded to aromatic compound C=C–C stretch, 1419.61 corresponded to vinyl C–H plane bend of the olefinic compound, 1363.67 corresponded to tertiary alcohol O–H bend, 904.61 indicated the silicate ion, 839.03 corresponded to C–H stretch, 522.71 indicated the aliphatic-iodo compound of C–I stretch and 435.91 indicated the thiols and thio-substituted compound of S–S stretch. Thus, the aromatic ring and peak at 522.71 was the specific absorption of the ZnO bond (Figure 3). In contrast, the broad absorption peak at 3483 was the characteristic absorption of the hydroxyl presence in the FTIR band that was responsible for ZnONP production.

#### 3.2.3. X-ray Diffraction Analysis of ZnONP

**Figure 4:**
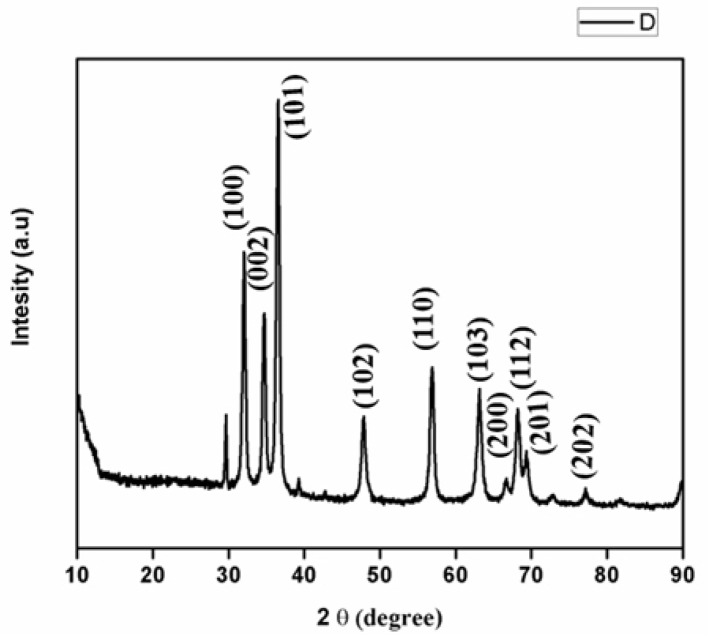
XRD analysis of ZnONP.

XRD investigation was used to confirm the crystal structure and size of the nanoparticles. The XRD peak determined the average crystallite sizes for the ZnO, peak 101 at 36° and 110 at 56°. The peaks (Figure 4) were assigned to the diffraction signals of 31.8° (100), 34.4° (002), 36.2° (101), 47.6° (102), 62.9° (103), 66.3° (200), 69.2° (201). The crystalline structure of the produced ZnONP nanocrystals matched the gold standard as determined by JCPDS (file no. 01-080-0074). A strong X-ray scattering centre in the crystalline phase could be attributable to the capping agents [42], according to intense Bragg reflections. The process of centrifugation and redispersion of the pellet in Millipore water after nanoparticle formation as part of the purification process proved the independent crystallization of capping agents. As a result, the XRD results revealed that bio-organic phase crystallization occurs on the surface of zinc nanoparticles or vice versa [43]. The broadening of peaks in solid XRD patterns is typically cited as the cause of the particle size effect. Smaller particle sizes and the effects of the experimental conditions on crystal nuclei nucleation are indicated by wider peaks. The primary peaks were (101) 36° and (110) 57°, respectively, and the nanoparticle size ranged from 100 to 200 nm.

#### 3.2.4. Dynamic Light Scattering (DLS) and ζ-Potential of ZnONP

**Figure 5:**
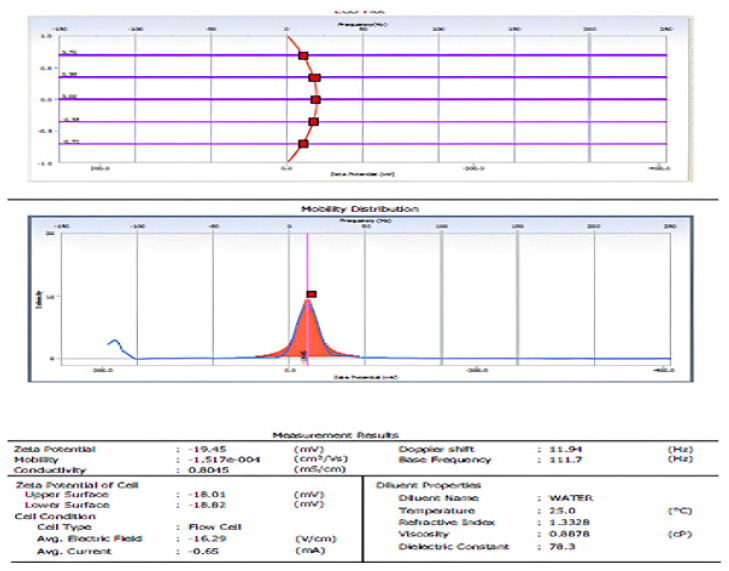
ζ-potential analysis of ZnONP.

The particle size was calculated using a dynamic light scattering technique that used Brownian motion in a colloidal solution to assess particle size. This approach was used to determine the average particle size, size distribution, and polydispersity index (PDI) of synthesized ZnONP [44]. The resulting ZnO particles had a size distribution ranging from 78 to 145 nm. It had an average particle size of 145.1 nm (Figure 5).

The ζ-potential study was performed to determine the surface charge obtained by ZnONP, which can be used to learn more about the stability of the colloidal ZnONP that was synthesized. Potential values greater than +30 mV or less than −30 mV are generally regarded to result in a stable suspension [24]. In water as a dispersant at 25 °C, the ζ-potential of the produced ZnONP was measured. The results with diluent properties of viscosity 0.8878 cP and dielectric constant of 78.3, 1.3328 referencing index. The ζ-potential as −19.45 mV indicated the stability of the nanoparticle, mobility −1.517 × 10^−4^ cm^2^/Vs, and conductivity 0.8045 mS/cm.

#### 3.2.5. TEM

**Figure 6:**
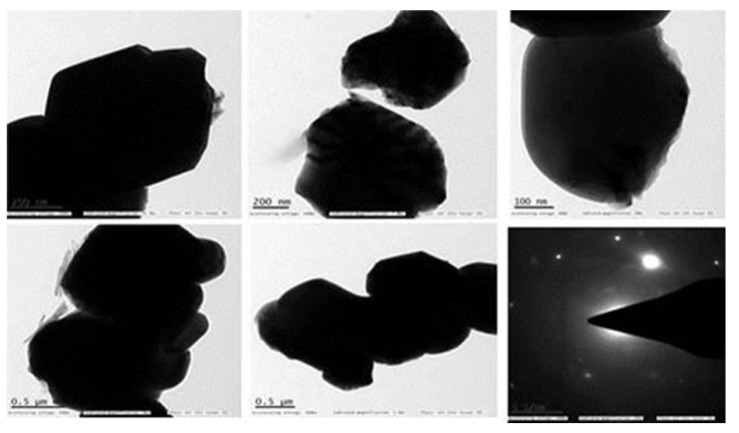
TEM analysis of ZnONP.

The morphology and size of the produced ZnONP, as well as their related SAED patterns and lattice characteristics, were validated by a high-resolution TEM examination. The higher 0.5 μm and lower 100 nm magnifications of the TEM pictures exhibited in Figure 6 showed that the ZnONP had been produced. Most nanoparticles have a spherical form with an agglomerated surface due to high-density growth [45]. TEM has been used to evaluate the shape and size of the resulting particles.

#### 3.2.6. FESEM and EDX Analysis

**Figure 7:**
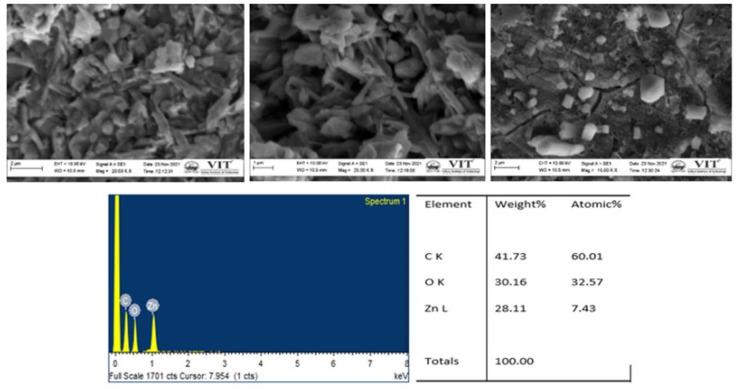
SEM and EDX analysis of ZnONP.

The surface morphology of the ZnONP was depicted in FESEM pictures. A typical SEM investigation of ZnONP synthesized from *C. cuspidatus* leaf extract is shown in Figure 7. ZnONP crystallites are spherical with a needle shape distribution, which correlates well with crystallite size determined by XRD. Because of the presence of biomolecules in the leaf, there is a little aggregation of nanoparticles. Stabilized nanoparticles were encapsulated from each other by the stabilising agent and could be redispersed. The surfactant was critical in determining particle size distribution and preventing clustering and flocculation. Furthermore, the ZnONP had a flat surface due to the green production procedure. EDX was used to conduct more research on ZnONP. The EDX equipment validated the detection of a finite number of elements, indicating that the ZnONP was composed solely of zinc, carbon, and oxygen, with no visible impurities [41,46]. The purity of ZnONP was demonstrated by all of the exhibited peaks attributed to Zn and 30% oxygen, explaining that the synthesized nanoparticle was in purified form without any unknown indications.

#### 3.2.7. Atomic Force Microscopy (AFM) Analysis

**Figure 8:**
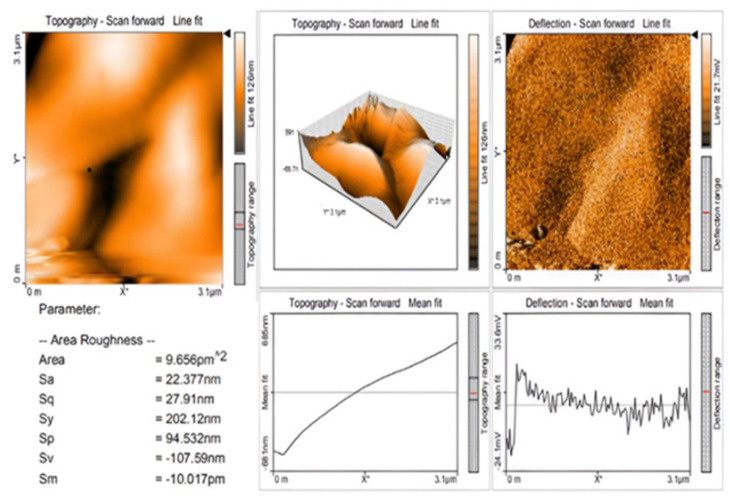
AFM image of ZnO nanoparticles biosynthesized using of *C. cuspidatus leaf* extract.

Using the AFM software’s roughness analysis option, the root mean square (RMS) roughness (Rq) parameter was calculated for the entire image. Figure 8 displays the AFM topography and 3D pictures of the ZnONP thin film produced on the glass substrate. This parameter indicates the standard deviation of the surface height values inside a specific area [47]. ZnONP RMS (Rq) values for 3 × 3 μm^2^. The ZnONP sample’s RMS (Rq) value was 27.91 nm, while its RMS (Ra) value was 22.377 nm. Similarly, these results also showed the 3D AFM image of ZnO NPs using line profile. The result of line profile images of ZnO NPs depicted NPs of size < 50 nm. The XRD results also confirmed the NPs size [48]. The polydispersed ZnO NPs size measured by AFM 3D image was in the range of 20–55 nm.

#### 3.2.8. X-ray Photoelectron Spectroscopy (XPS) Analysis

**Figure 9:**
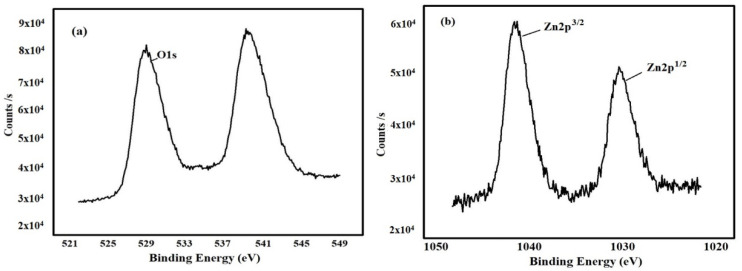
The X-ray photoelectron spectroscopy for ZnONP synthesized suing *C. cuspidatus*, (**a**) O1s binding energy area (**b**) Zn2p binding area.

In Gaussian fitting, XPS spectra of ZnONP showed asymmetric O (1s) signals containing three symmetrical signals, namely O1s1, O1s2 and O1s3 (Figure 9). The *C. cuspidatus* leaf extracts synthesized ZnONP results showed three signals O1s1 at 531.49 eV, due to the O^2−^ ion in Zn^2+^ ion. The low binding energy component located at 530.9 ± 0.1 eV was attributed to O^2−^ ions participating in the Zn–O bond in the wurtzite structure of the hexagonal Zn^2+^ ions of ZnO [49]. The high binding energy component located at 532.7 ± 0.7 is reported to be associated with oxygen species adsorbed on the surface of the ZnO, such as –CO_3_, H_2_O, or O_2_ [50]. These results were evidenced by Armelao et al. [50], who reported adsorbed H_2_O and lattice oxygen on the ZnO surface, Zn–O–Zn surface and Zn–OH surface, respectively.

### 3.3. Antioxidant Activity

**Figure 10:**
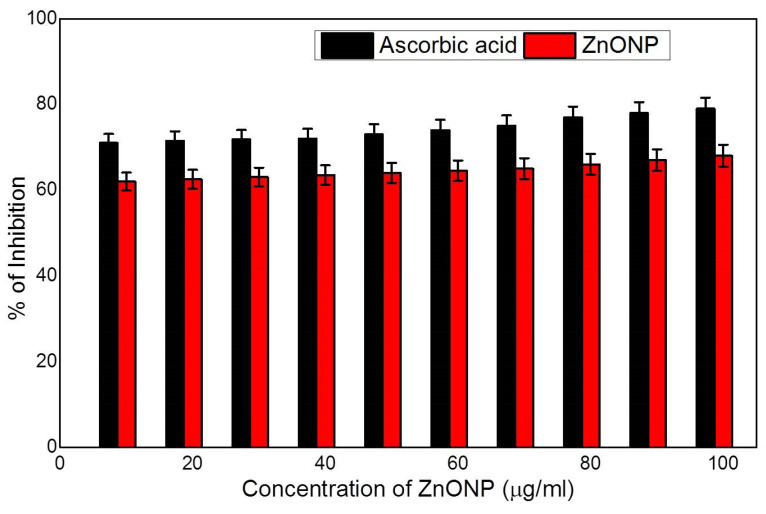
Antioxidant activity of ZnONP.

Antioxidants are molecules that shield cells from the damaging effects of reactive oxygen species (ROS). Natural antioxidants have been progressively prevalent due to their ability to prevent diseases and maintain cell balance. Natural antioxidants are essential for scavenging toxic free radicals and preventing cell oxidative damage. With its high redox potential, ZnONP may split water molecules into hydroxyl and hydrogen radicals, which can then be used to reduce free radicals and stabilise DPPH molecules. To measure the antioxidant property, sonicated ZnONP was used in water as a dispersant at 25 °C. In the DPPH assay, different concentrations are dosage independent. The unique antioxidant effects of zinc have been demonstrated by numerous reporters [51]. As a result, the current work used the DPPH assay to determine the antioxidant activity of ZnONP. Figure 10 shows an increase in ZnONP content with very few variations. We observed that ZnONP showed an inhibition rate of 70% at 5 to 60 μg/mL and above 70% at 65 to 100 μg/mL. Due to the presence of metal nanoparticles, the results of ZnONP and ascorbic acid were practically identical.

### 3.4. Anticancer Activity in MCF-10A, MCF-7, and A549 Cells Studied by MTT Assay

**Figure 11:**
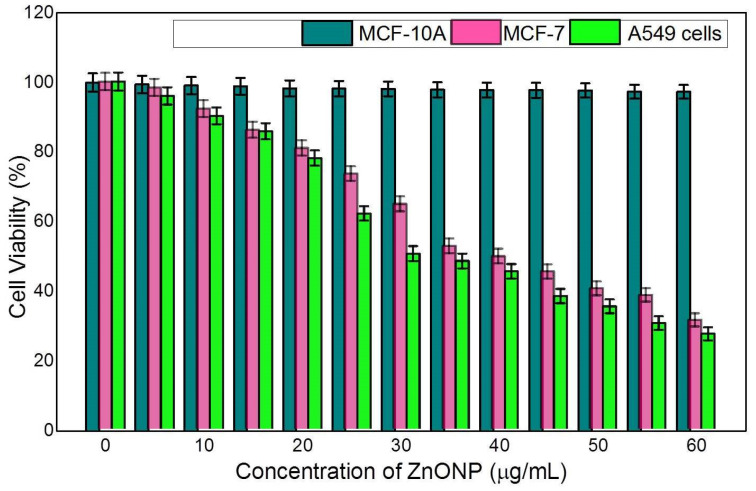
Cytotoxicity activity of ZnONP in MCF-10A, MCF-7 and A549 cell lines.

Finding medication to treat different types of cancer is difficult since it is a severe threat to public health. ZnONP toxicological effectiveness has been analyzed in normal (MCF-10A) and cancerous cell lines, namely, MCF-7 and A549. According to several researchers, ZnONP has no cytotoxicity effect on normal cells (MCF-10A) and is exclusively hazardous to malignant cells. The results showed that exposing MCF-7 and A549 cells to different concentrations of ZnONP for 72 h significantly reduced the cell viability, except at the higher concentration of 60 μg/mL. As the concentration increased, the cell survivability decreased considerably (Figure 11). When MCF-7 cells were exposed to ZnONP from 5 to 37 μg/mL, the cell viability decreased significantly from 98.32 to 31.37%. The cell viability of A549 decreased from 95.85 to 27.35% when the concentration was increased from 5 to 30 μg/mL. However, ZnONP does not have a major impact on the cell viability of normal cells, MCF-10A. ZnONP-mediated cytotoxicity may result from the interaction between a ligand and a receptor, which activates a signalling pathway. The viability of treated cell lines was dose-dependent, according to data analysis. With the use of our findings, earlier research validated that ZnONP has an appositive impact on cancerous cells [52,53]. Previously, several studies have shown that ZnONP exhibited potential cytotoxicity toward various types of cancer cells. Moreover, it was suggested that the improved permeability and retention activity and electrostatic interaction properties of ZnONP as well as the enhanced ROS in cancer cells may facilitate the ZnONP-mediated enhanced cytotoxicity in cancer cells [54,55]. In line with these studies, the cytotoxicity induced by photosynthesized ZnONP is probably due to Zn-associated protein activity disequilibrium and oxidative stress via ROS generation. Furthermore, phytoconstituents present in the plant extracts were also shown to possess anticancer activity. For example, the potent cytotoxic effect of ZnONP synthesized using *Vinca rosea* leaves extract against the MCF7 breast cancer cell line was reported, and this is due to the alkaloids in extracts which induce oxidative stress and lead to cell death or damage [56,57].

## 4. Conclusions

The rapid biosynthesis of ZnONP utilizing *C. cuspidatus* leaf extract is an eco-friendly and straightforward nanoparticle synthesis. ZnONP was characterized using UV–Vis, FTIR, XRD, SEM, EDX, TEM, AFM and XPS analysis. The SEM image revealed a spherical nanoparticle with a diameter ranging from 100 to 200 nm. FTIR showed that a peak at 522.71 cm^−1^ was a characteristics absorption of the ZnO bond, indicating a hexagonal wurtzite phase, which is a stable form of the ZnO under ambient conditions. The size distribution of nanoparticles was indicated by the particle size of 145.1 nm −19.45 mV was discovered to be the ζ-potential. The anticancer efficacy of ZnONP on MCF-7 and A549 cell lines was investigated in this work. The findings showed that conditional chemotherapeutic drugs using ZnONP could have a wider variety of applications in treating cancer cells. This study also revealed ZnONP’s biological properties, including its strong antioxidant and anticancer potential. The potency of ZnONP was shown to be dose-dependent in the research. More in vitro, in vivo, and molecular analysis in animal models is needed in the future to assess their nano-pharmacological relevance in various bioactivities.

## Data Availability

Not applicable.
